# Tranexamic acid in pertrochanteric fractures: a retrospective analysis of perioperative outcomes after fixation with a proximal femoral nail

**DOI:** 10.1186/s12891-022-05889-3

**Published:** 2022-11-03

**Authors:** John Hanke, Thomas Mendel, Matthias Wingert, Philipp Schenk, Markus Heinecke, Arne Wilharm

**Affiliations:** 1 Department of Trauma and Reconstructive Surgery, BG Klinikum Bergmannstrost Halle gGmbH, Merseburger Strasse 165, 06120 Halle (Saale), Germany; 2 Department of Trauma-, Hand- and Reconstructive Surgery, University Hospital Jena, Am Klinikum 1, 07747 Jena, Germany; 3 Research Executive Department, BG Klinikum Bergmannstrost Halle gGmbH, Merseburger Strasse 165, 06120 Halle (Saale), Germany

**Keywords:** Tranexamic acid, Proximal femoral fracture, Haemoglobin monitoring, Geriatric, Proximal femur nail

## Abstract

**Background:**

Treatment of pertrochanteric femoral fractures is often associated with significant blood loss. It has already been demonstrated that the administration of tranexamic acid (TXA) for endoprosthetic procedures reduces blood losses and leads to a decreased frequency of postoperative complications. The aim of this study is to demonstrate whether the administration of TXA as part of osteosynthesis treatment for pertrochanteric fractures using a proximal femoral nail reduces perioperative blood losses and haemorrhage-related complications.

**Methods:**

In a two-centre retrospective cohort study, 1 g TXA i.v. was administered preoperatively to 294 patients who had suffered from pertrochanteric femoral fractures. The subjects were compared clinically to a historical control group who did not receive TXA (nonTXA). Outcomes were evaluated on the basis of perioperative blood loss, transfusion requirement, and occurrence of complications.

**Results:**

The TXA group showed evidence of a reduction in blood loss (TXA = 0.97 ± 0.47 l; nonTXA = 1.06 ± 0.47 l; *p* = 0.004) and a lower frequency of transfusion (TXA = 20%; nonTXA = 31%; *p* = 0.032) as compared to the nonTXA group. However, evidence of this therapeutic effect could only be demonstrated at one of the centres on subgroup comparison between the two centres. At the second centre, the data did not show a significant difference. A trend could be seen towards a reduction in postoperative renal failure. No complications occurred resulting from the administration of tranexamic acid.

**Conclusion:**

Preoperative administration of TXA does not lead to an increased rate of thromboembolic complications when applied for treatment of pertrochanteric femoral fractures. Evidence of a positive effect could be seen in principle in relation to the reduction in perioperative blood loss and the frequency of transfusion. The difference in effect between the two centres remains to be clarified: for this reason, it is possible to assume that further factors influencing the efficacy of TXA administration are at play which were not taken into account in this study.

## Background

Pertrochanteric femoral fractures are the second most common geriatric fractures in Germany in people over 70 years of age, with an annual incidence of 486 per 100,000 inhabitants, second only to medial neck-of-femur fractures [1]. They not only pose a significant risk to the patients affected in terms of their independence to carry out activities of daily living [2], but can also be life-threatening. For example, the 1-year mortality rate following hip fractures is almost 30% [3].

Approximately 82% of hip-joint fractures treated in Germany with an osteosynthesis approach are now stabilised using an intramedullary nail [4]. In addition to the fracture event itself, both the surgical procedure and any associated blood loss represent a significant burden for elderly patients, who tend to have multiple morbidities. From the literature, rates of perioperative transfusion requirement range from 30% to 84.6% depending on the type of fracture and level of invasiveness [5–9]. These data from the literature indicate a mean blood loss of up to 2100 ml [9]. Blood loss in cases of intracapsular fracture is lower than in extracapsular fracture cases [10].

Postoperative anaemia following treatment of hip fractures leads to greater difficulties in mobilising the patient, increased duration of in-patient treatment, and increased mortality [11, 12]. It should also be noted that blood transfusions can also increase postoperative mortality and morbidity [13]. Due to immune system impairment, the incidence of wound infections and cardiac complications increases, resulting in rising treatment costs [14–16].

In this respect, preventive efforts are aimed at minimising perioperative blood loss. A promising approach can be seen in the use of tranexamic acid (TXA). TXA is a cost-effective synthetic derivative of the amino acid lysine: it binds reversibly to plasminogen to produce an anti-fibrinolytic effect [17]. It has been used since 1966 in various different forms for the treatment of bleeding. Today, TXA is used as part of standard care for elective hip replacement procedures in many hospitals. It has been demonstrated that its use has led to a significant reduction in blood transfusion requirement as well as frequency of renal failure with no increase in complications [18, 19]. Similar results have also been obtained in the area of spinal surgery [20].

From the literature, the first indications can be seen of a similar positive effect of TXA used in the treatment of proximal femoral fractures. For example, Sadegi et al. (2007) saw a reduction in measured blood loss from an average of 1,484 ml to 960 ml due to administration of TXA in patients receiving treatment for hip fractures [5]. A problematic aspect of previous studies was that the number of cases tended to be small. Additionally, different osteosynthesis approaches, and in some cases even endoprosthetic treatments, were included in the studies [5, 21, 22]. In 2016, Farrow et al. published an initial meta-analysis of the available literature, whereby only five publications could be included [23]. The diversity of the studies with regard to the fracture types and surgical treatment approaches included (indeed, these were not even indicated for all studies), makes it difficult to carry out a direct comparison. Particularly striking was the inclusion of some cases with protracted operating times of over 2 h [5, 9, 21, 22, 24, 25].

In summary, Farrow et al. concluded, with a moderate level of evidence, that the administration of TXA reduced the amount of blood transfusions required during treatment of hip fractures [23].

### Study objectives

The aim of this study is to determine, on the basis of a homogeneous study population, whether preoperative intravenous (i.v.) administration of TXA as part of treatment of pertrochanteric femoral fractures with an intramedullary nail can lead to a reduction in blood loss and frequency of transfusion. The secondary objective of the study is to determine whether the incidence of postoperative complications, such as acute kidney failure, heart attack and death, as well as duration of postoperative admission decreases following administration of TXA. Furthermore, the frequency of TXA-associated complications (thrombosis, embolism, stroke, seizures) is to be recorded.

## Methods

### Study design

Based on the positive results obtained in the field of elective hip and knee replacements [19, 26], preoperative i.v. administration of TXA has been applied since 2016 as part of surgical treatment for pertrochanteric femoral fractures with an intramedullary nail at two level-1 trauma centres in accordance with the internal standard operating procedure (SOP). In a two-centre retrospective case–control study, the cohorts of one group of TXA patients (TXA group) in each of the two hospitals were compared to one consecutive group of patients respectively whose surgical treatment took place prior to 2016, i.e. before the introduction of the SOP for preoperative TXA administration (nonTXA group). To ensure better comparability, these groups only included patients who had no contraindications for administration of TXA.

**Fig. 1:**
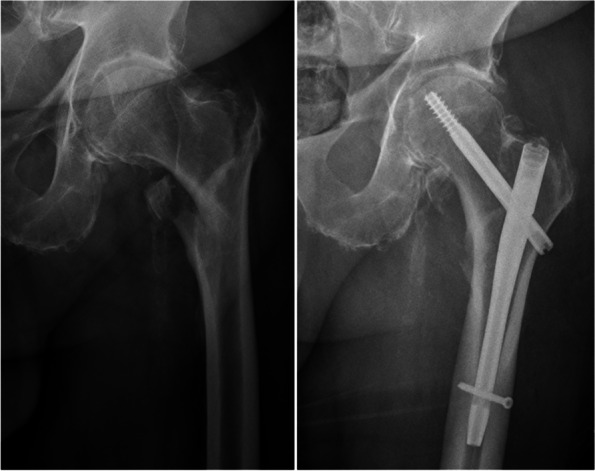
Pertrochanteric femoral fracture and osteosynthesis with an intramedullary nail (Stryker, Gamma III)

Following recommendations from the literature [25], in the TXA group of each hospital respectively the standard dose of 1 g TXA i.v. was administered 10 min preoperatively once any contraindications had been ruled out and with doses adjusted according to the specialist guidelines in any cases of renal insufficiency (Table 1). Presentation of a pertrochanteric femoral fracture of types 31-A1 to A3 according to the AO classification was defined as the primary inclusion criterion (Fig. 1). Both minimally-invasive and open surgical approaches were included in the analysis. Presentations with any further musculoskeletal injuries were excluded. In addition, those patients having taken any anticoagulant other than acetylsalicylic acid were excluded. Patients in the TXA group were operated on between 11/2016 and 12/2019. The nonTXA group received treatment between 9/2015 and 10/2016.

**Table 1:** Overview of tranexamic acid administration (indication, contraindication, adjustments in renal insufficiency)

**Indication for the administration of tranexamic acid**
All hip fractures
**Contraindications to the administration of tranexamic acid**
Known intolerance
Severe renal insufficiency (risk of accumulation; see below)
Congenital or acquired thrombophilia
Acute arterial and venous thrombosis
Patient history of arterial/venous thrombosis or ischaemic stroke
History of stent implantation under dual platelet inhibition
Known epilepsy
Pregnancy and lactation
Hyperfibrinolysis as a result of disseminated intravascular coagulopathy
Bleeds in the urinary tract
Patients taking oral contraceptives (increased thrombogenic risk)
**Adjustments in case of renal insufficiency**
Serum creatinine 120 to 249 μmol/l: 10 mg/kg body weight (no further administration for the next 12 h)
Serum creatinine 250 to 500 μmol/l: 10 mg/kg body weight (no further administration for the next 24 h)

### Data Collection

Through analysis of the digital medical records, it was possible to record the following data for all patients: demographic data (gender, age, weight, height, body mass index (BMI), aspirin intake, fracture type, ASA score); process parameters (surgical technique, knife-to-skin time, drainage system, duration of postoperative admission); and complications (thrombosis, embolism, stroke, heart attack, seizure, death, other); laboratory data from the day of admission (haemoglobin (Hb), haematocrit (Hct), platelet count, creatinine (Crea), glomerular filtration rate (GFR), quick time, activated partial thromboplastin time (aPTT); and from 1st postoperative day (Hb, Hct, platelet count); as well as the lowest GFR or highest creatinine values recorded over the course of treatment. Furthermore, all instances of administration of red cell concentrate (RCC) were recorded.

Preoperative blood volume could be determined by applying the formula devised by Nadler et al. [27], and blood loss determined following the method of Good et al. [28].

### Statistical Analysis

Firstly, analysis was performed for each site separately (Clinic 1: University Hospital Jena and Clinic 2: BG Klinikum Bergmannstrost Halle) as well as across both locations (overall) to determine whether there were differences between the patient groups with respect to the demographic data, fracture morphology (AO classification) and the duration of the preoperative in-patient admission. For this purpose, a Chi^2^ test and multivariate analysis of variance (MANOVA) were applied.

In addition, MANOVA was used to test whether there were significant differences in the laboratory parameters recorded between the patient groups preoperatively.

The effects of administration of TXA on the primary outcome parameters of blood loss and need for RCC administration were analysed using Chi^2^ and U tests.

With respect to the secondary outcome parameters, the duration of postoperative in-patient admission and frequency of renal failure and death were compared using a Chi^2^ test.

SPSS V.26 (IBM SPSS Statistics for Windows. Armonk, NY: IBM Corp.) was used for the statistical analysis. The threshold for significance was set at *p* = 0.05.

## Results

Two hundred ninety-four patients across two sites (Clinic 1: *N* = 138, Clinic 2: *N* = 156) were included in the study. On average, the patients (79 men, 215 women) were 81 ± 11 years old. The BMI averaged 26 ± 5 kg/m^2^. On average, the surgical treatment was provided 0.6 days following admission. No differences could be identified between either the TXA and nonTXA group, nor between the sites, with respect to these parameters (Table 2).

**Table 2:** Preoperative parameters, description of the study population and their mean values, standard deviations (mean ± SD) as well as minimum and maximum values (range). The values are presented both overall across groups as well as separately for patients treated with and without tranexamic acid (TXA) and for the two centres. The *p*-value (P) with respect to the differences between the two patient groups (nonTXA and TXA) and between the two centres (location) is listed accordingly

	**overall**				**Clinic 1 **				**Clinic 2**				**location**
	total	nonTXA	TXA		total	nonTXA	TXA		total	nonTXA	TXA		total
	(*N* = 294)	(*N* = 147)	(*N* = 147)	*p*-value	(*N* = 138)	(*N* = 69)	(*N* = 69)	*p*-value	(*N* = 156)	(N = 78)	(N = 78)	*p*-value	*p*-value
Sex, n (%)				0.599				0.442				0.104	1.000
Male	79 (27)	42 (29)	37 (25)		37 (27)	16 (23)	21 (30)		42 (27)	26 (33)	16 (21)		
Female	215 (73)	105 (71)	110 (75)		101 (73)	53 (77)	48 (70)		114 (73)	52 (67)	62 (79)		
Age, y				0.543				0.846				0.293	0.337
Mean (SD)	81 ± 11	81 ± 11	81 ± 12		80 ± 11	81 ± 11	80 ± 10		82 ± 12	81 ± 11	83 ± 13		
Range	33–102	48–97	33–102		42–96	51–96	42–95		33–102	48–97	33–102		
Body weight, kg				0.823				0.996				0.749	0.380
Mean (SD)	69 ± 15	69 ± 15	69 ± 16		70 ± 15	70 ± 13	70 ± 17		69 ± 15	68 ± 17	69 ± 14		
Range	38–118	38–115	39–118		45–118	46–100	45–118		38–115	38–115	39–107		
Body height, m				0.832				0.736				0.489	0.465
Mean (SD)	1.64 ± 0.09	1.65 ± 0.09	1.64 ± 0.09		1.65 ± 0.1	1.65 ± 0.09	1.65 ± 0.1		1.64 ± 0.09	1.65 ± 0.1	1.64 ± 0.09		
Range	1.44–2.00	1.48–1.91	1.44–2.00		1.44–2.00	1.48–1.91	1.44–2.00		1.47–1.90	1.48–1.86	1.47–1.90		
BMI, kg/m^2^				0.706				0.653				0.342	0.631
Mean (SD)	26 ± 5	25 ± 5	26 ± 5		26 ± 5	26 ± 4	26 ± 5		25 ± 5	25 ± 5	26 ± 5		
Range	15–40	15–38	16–40		18–38	19–38	18–36		15–40	15–38	16–40		
ASS, n (%)				0.715				1.000				0.745	**0.020**
Yes	104 (35)	50 (34)	54 (37)		39 (28)	19 (27)	20 (29)		65 (42)	31 (40)	34 (44)		
No	190 (65)	97 (66)	93 (63)		99 (72)	50 (73)	49 (71)		91 (58)	47 (60)	44 (56)		
AO classification, n (%)				0.785				0.975				0.708	**0.002**
A1	111 (38)	55 (37)	56 (38)		60 (44)	29 (42)	31 (45)		51 (33)	26 (33)	25 (32)		
A2	125 (43)	65 (44)	60 (41)		44 (31)	23 (33)	21 (30)		81 (52)	42 (54)	39 (50)		
A3	58 (20)	27 (18)	31 (21)		34 (25)	17 (25)	17 (25)		24 (15)	10 (13)	14 (18)		
ASA				0.441				0.759				0.405	**0.004**
Mean (SD)	2.7 ± 0.5	2.8 ± 0.5	2.7 ± 0.6		2.6 ± 0.6	2.7 ± 0.5	2.6 ± 0.6		2.8 ± 0.5	2.9 ± 0.4	2.8 ± 0.5		
Range	1–4	2–4	1–4		1–4	2–4	1–4		1–4	2–4	1–4		
Hb, mmol/l				**0.002**				0.153				**0.004**	0.319
Mean (SD)	7.83 ± 0.98	8.01 ± 0.98	7.66 ± 0.95		7.89 ± 0.93	8 ± 0.93	7.78 ± 0.92		7.78 ± 1.02	8.01 ± 1.02	7.55 ± 0.98		
Range	4.5–10.5	4.5–10.5	5.1–9.7		5.5–10.3	06.10.2003	5.5–9.7		4.5–10.5	4.5–10.5	5.1–9.7		
HK, %				** < 0.001**				**0.040**				**0.003**	0.473
Mean (SD)	0.38 ± 0.05	0.39 ± 0.05	0.37 ± 0.05		0.38 ± 0.04	0.39 ± 0.04	0.37 ± 0.04		0.38 ± 0.05	0.39 ± 0.05	0.36 ± 0.05		
Range	0.22–0.51	0.22–0.51	0.25–0.47		0.26–0.49	0.29–0.49	0.26–0.47		0.22–0.51	0.22–0.51	0.25–0.47		
pre-surgery stay, (d)				0.249				0.591				0.253	0.893
Mean (SD)	0.6 ± 0.7	0.7 ± 0.8	0.5 ± 0.7		0.6 ± 0.8	0.6 ± 0.9	0.6 ± 0.6		0.6 ± 0.7	0.7 ± 0.7	0.5 ± 0.7		
Range	0–7	0–7	0–2		0–7	0–7	0–2		0–3	0–3	0–2		

Patients from Clinic 2 had a slightly higher ASA score on average (Clinic 1: 2.6 ± 0.6, Clinic 2: 2.8 ± 0.5; *p* = 0.004) and took aspirin more frequently (Clinic 1: 28%, Clinic 2: 42%; *p* = 0.020). However, there were no significant differences for these parameters between the study groups at the respective sites (Table 2).

Preoperatively, the TXA group in Clinic 2 had a lower Hb, and at both sites this group had a lower Hct value than the nonTXA group, such that these laboratory values showed differences across the two groups preoperatively (preOP Hb: NonTXA 8.01 ± 0.98, TXA 7.66 ± 0.95; *p* = 0.002; preOP Hct: NonTXA 0.39 ± 0.05, TXA 0.37 ± 0.05, *p* < 0.001) (Table 2).

The majority of the fractures treated were A2 fractures (43%), followed by A1 fractures (38%) and A3 fractures (20%) (Table 2).

Surgical stabilisation using an intramedullary nail was carried out in 254 patients (86%) using a closed (CRIF) approach, and in 40 patients (14%) through open reduction and internal fixation (ORIF) (Table 3). The knife-to-skin times between the nonTXA and TXA groups only differed in Clinic 2 between the nonTXA and the TXA groups treated with CRIF, by an average of 8 min. There were no significant differences between the sites in this respect (Table 3).

**Table 3:** Intraoperative parameters, surgical approach and knife-to-skin time, mean values, standard deviations (mean ± SD) as well as minimum and maximum values (range). The values are presented both overall across groups as well as separately for patients treated with and without tranexamic acid (TXA) and for the two centres. The *p*-value (P) is with respect to the differences between the two patient groups (nonTXA and TXA) and between the two centres (location) is listed accordingly

	**overall**				**Clinic 1**				**Clinic 2**				**Location**
	total	nonTXA	TXA		total	nonTXA	TXA		total	nonTXA	TXA		total
	(*N* = 294)	(*N* = 147)	(*N* = 147)	*p*-value	(*N* = 138)	(*N* = 69)	(*N* = 69)	*p*-value	(*N* = 156)	(*N* = 78)	(N = 78)	*p*-value	*p*-value
Surgery technique, n (%)				1.000				1.000				1.000	0.308
ORIF	40 (14)	20 (14)	20 (14)		22 (16)	11 (16)	11 (16)		18 (12)	9 (12)	9 (12)		
CRIF	254 (86)	127 (86)	127 (86)		116 (84)	58 (84)	58 (84)		138 (88)	69 (88)	69 (88)		
Cut suture time, (min)				0.649				0.082				0.252	0.655
Mean (SD)	60 ± 32	60 ± 34	61 ± 30		61 ± 34	66 ± 37	56 ± 30		59 ± 31	56 ± 32	62 ± 29		
Range	18–225	18–225	21–165		18–185	18–185	21–155		20–225	20–225	23–140		
ORIF				0.111				0.066				0.677	0.981
Mean (SD)	110 ± 42	122 ± 47	100 ± 35		111 ± 40	126 ± 41	95 ± 34		111 ± 46	116 ± 55	106 ± 37		
Range	31–225	41–225	31–155		42–185	74–185	42–155		31–225	41–225	31–140		
CRIF				0.605				0.149				**0.025**	0.702
Mean (SD)	52 ± 21	51 ± 20	52 ± 23		51 ± 22	54 ± 22	48 ± 23		52 ± 20	48 ± 17	56 ± 22		
Range	18–130	18–112	21–130		18–123	18–112	21–123		20–130	20–101	23–130		

At both sites, TXA was administered to 50% of patients treated using CRIF and ORIF (Table 3).

**Fig. 2:**
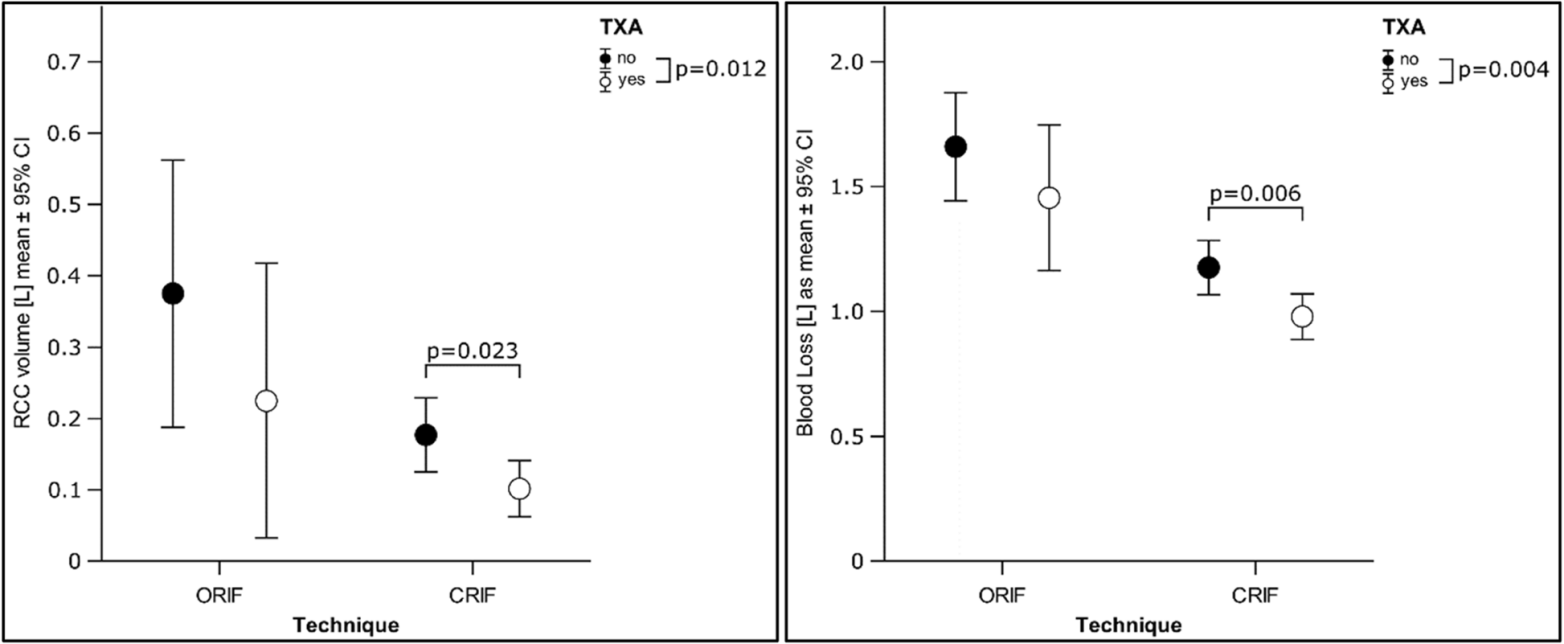
Mean values and 95%-confidence intervals are shown for the open and percutaneous approaches for blood loss and transfusion volume taking into account administration of TXA

Administration of TXA in Clinic 1 led to a significant reduction in blood loss in both the open (non TXA = 1.25 ± 0.36 l, TXA = 1.02 ± 0.34 l; *p* = 0.034) and the percutaneous (nonTXA = 0.97 ± 0.47 l, TXA = 0.79 ± 0.39 l; *p* = 0.001) approaches; whilst blood loss in Clinic 2 showed no differences between TXA and nonTXA groups (ORIF: nonTXA = 1.64 ± 0.45 l, TXA = 1.30 ± 0.50 l; *p* = 0.499; CRIF: nonTXA = 1.06 ± 0.42; TXA = 1.06 ± 0.50 l; *p* = 0.567). However, the reduction in blood loss was so pronounced in Clinic 1 that a significant reduction in blood loss was also seen when taking an overall view both for patients treated with CRIF (nonTXA = 1.01 ± 0.44 l, TXA = 0.94 ± 0.47 l; *p* = 0.06) and for all patients (nonTXA = 1.06 ± 0.47 l, TXA = 0.97 ± 0.47 l; *p* = 0.004) (Table 4, Fig. 2).

**Table 4:** Blood transfusions (RCC = red cell concentrate) and total blood losses, mean values, standard deviations (mean ± SD), and minimum and maximum values (range). The values are presented both overall across groups as well as separately for patients treated with and without tranexamic acid (TXA) and for the two centres. The *p*-value (P) is with respect to the differences between the two patient groups (nonTXA and TXA) and between the two centres (location) is listed accordingly

	**overall**				**Clinic 1**				**Clinic 2**				**Location**
	total	nonTXA	TXA		total	nonTXA	TXA		total	nonTXA	TXA		total
	(*N* = 294)	(*N* = 147)	(*N* = 147)	*p*-value	(*N* = 138)	(*N* = 69)	(*N* = 69)	*p*-value	(*N* = 156)	(*N* = 78)	(*N* = 78)	*p*-value	*p*-value
RCC volume, (l)				**0.012**				** < 0.001**				0.623	0.796
Mean (SD)	0.13 ± 0.24	0.17 ± 0.26	0.1 ± 0.22		0.13 ± 0.25	0.22 ± 0.29	0.04 ± 0.15		0.14 ± 0.24	0.13 ± 0.24	0.15 ± 0.25		
Range	0–1	0–1	0–1		0–1	0–1	0–1		0–1	0–1	0–1		
ORIF				0.250				0.066				0.730	0.356
Mean (SD)	0.25 ± 0.34	0.31 ± 0.33	0.19 ± 0.34		0.3 ± 0.35	0.43 ± 0.34	0.16 ± 0.32		0.19 ± 0.33	0.17 ± 0.28	0.22 ± 0.38		
Range	0–1	0–1	0–1		0–1	0–1	0–1		0–1	0–0,75	0–1		
CRIF				**0.023**				** < 0.001**				0.713	0.261
Mean (SD)	0.12 ± 0.22	0.15 ± 0.25	0.08 ± 0.19		0.1 ± 0.21	0.18 ± 0.26	0.02 ± 0.08		0.13 ± 0.23	0.12 ± 0.23	0.14 ± 0.23		
Range	0–1	0–1	0–0,75		0–1	0–1	0–0,5		0–1	0–1	0–0,75		
RCC, n (%)				**0.032**				** < 0.001**				0.716	0.789
Yes	75 (25)	46 (31)	29 (20)		34 (25)	27 (39)	7 (10)		41 (26)	19 (24)	22 (28)		
No	219 (75)	101 (69)	118 (80)		104 (75)	42 (61)	62 (90)		115 (74)	59 (76)	56 (72)		
ORIF				0.200				0.086				1,000	0.348
Yes	17 (43)	11 (55)	6 (30)		11 (50)	8 (73)	3 (27)		6 (33)	3 (33)	3 (33)		
No	23 (58)	9 (45)	14 (70)		11 (50)	3 (27)	8 (73)		12 (67)	6 (67)	6 (67)		
CRIF				0.100				**0,001**				0.696	0.368
Yes	58 (23)	35 (28)	23 (18)		23 (20)	19 (33)	4 (7)		35 (25)	16 (23)	19 (28)		
No	196 (77)	92 (72)	104 (82)		93 (80)	39 (67)	54 (93)		103 (75)	53 (77)	50 (72)		
Blood loss (BL), (l)				**0.004**				** < 0.001**				0.766	0.924
Mean (SD)	1.01 ± 0.47	1.06 ± 0.47	0.97 ± 0.47		0.92 ± 0.43	1.02 ± 0.46	0.83 ± 0.39		1.09 ± 0.48	1.10 ± 0.47	1.09 ± 0.50		
Range	0.00–2.68	0.15–2.68	0.00–2.10		0.00–2.31	0.15–2.31	0.00–1.96		0.07–2.68	0.21–2.68	0.07–2.10		
ORIF				0.246				**0.034**				0.499	0.879
Mean (SD)	1.29 ± 0.45	1.42 ± 0.44	1.14 ± 0.43		1.14 ± 0.36	1.25 ± 0.36	1.02 ± 0.34		1.47 ± 0.49	1.64 ± 0.45	1.30 ± 0.50		
Range	0.39–2.43	0.62–2.43	0.39–2.10		0.39–1.76	0.62–1.76	0.39–1.51		0.54–2.43	0.92–2.43	0.54–2.10		
CRIF				**0.006**				**0.001**				0.567	0.616
Mean (SD)	0.97 ± 0.46	1.01 ± 0.44	0.94 ± 0.47		0.88 ± 0.44	0.97 ± 0.47	0.79 ± 0.39		1.05 ± 0.46	1.03 ± 0.42	1.06 ± 0.50		
Range	0.00–2.68	0.15–2.68	0.00–1.99		0.00–2.31	0.15–2.31	0.00–1.96		0.07–2.68	0.21–2.68	0.07–1.99

The proportion of patients who received a blood transfusion following surgery was significantly lower in the TXA group (nonTXA = 31%, TXA = 20%; *p* = 0.032) (Table 4). This difference can also be largely attributed to the patients in Clinic 1, where 39% of the nonTXA patients were transfused, falling to 10% for the TXA group (*p* =  < 0.001). In Clinic 2, on the other hand, no reduction in the frequency of transfusion could be identified (Table 4).

Twenty patients suffered from postoperative (postOP) kidney failure, whereby the majority of these patients underwent surgery in Clinic 1 (postOP kidney failure: Clinic 1 *N* = 18, Clinic 2 *N* = 2; *p* < 0.001). In the TXA group, the incidence was 4%, whilst 10% of patients in the nonTXA group suffered from postoperative renal failure. A significant difference could not be demonstrated here due to the small number of cases (Table 5). No further complications occurred in either centre.

**Table 5:** Postoperative parameters, mean values, standard deviations (mean ± SD) and minimum and maximum values (range). The values are presented both overall across groups as well as separately for patients treated with and without tranexamic acid (TXA) and for the two centres. The *p*-value (P) is with respect to the differences between the two patient groups (nonTXA and TXA) and between the two centres (location) is listed accordingly

	**overall**				**Clinic 1**				**Clinic 2**				**Location**
	total	nonTXA	TXA		total	nonTXA	TXA		total	nonTXA	TXA		total
	(*N* = 294)	(*N* = 147)	(*N* = 147)	*p*-value	(*N* = 138)	(*N* = 69)	(*N* = 69)	*p*-value	(*N* = 156)	(*N* = 78)	(*N* = 78)	*p*-value	*p*-value
Post-surgery stay, (d)				0.056				**0.007**				0.749	0.222
Mean (SD)	9 ± 3	9 ± 4	8 ± 3		9 ± 4	9 ± 4	8 ± 3		9 ± 3	9 ± 3	9 ± 3		
Range	3–27	3–27	3–16		3–27	3–27	3–16		3–17	4–17	3–16		
ORIF				0.172				0.062				0.441	0.900
Mean (SD)	9 ± 3	10 ± 4	9 ± 3		9 ± 4	11 ± 4	8 ± 3		10 ± 2	9 ± 2	10 ± 2		
Range	4–23	9–23	4–15		4–23	7–23	4–15		6–13	6–13	7–12		
CRIF				0.133				**0.03*9***				0.904	0.175
Mean (SD)	9 ± 3	9 ± 4	8 ± 3		8 ± 4	9 ± 4	8 ± 3		9 ± 3	9 ± 3	9 ± 3		
Range	3–27	3–27	3–16		3–27	3–27	3–16		3–17	4–17	3–16		
Renal failure, n (%)				0.103				0.075				1.000	** < *0.001***
Yes	20 (7)	14 (10)	6 (4)		18 (13)	13 (19)	5 (7)		2 (1)	1 (1)	1 (1)		
No	274 (93)	133 (91)	141 (96)		120 (87)	56 (81)	64 (93)		154 (99)	77 (99)	77 (99)		
dead, n (%)				1.000				1.000				-	0.102
Yes	3 (1)	2 (1)	1 (1)		3 (2)	2 (3)	1 (1)		0	0	0		
No	291 (99)	145 (99)	146 (1)		135 (98)	67 (97)	68 (99)		156 (100)	78 (100)	78 (100)		

The duration of postoperative admission was not significantly influenced by administration of TXA (*p* = 0.056) when both centres are considered together (Table 5). However, it appears that the CRIF patients in Clinic 1 who received TXA had significantly shorter postoperative admissions at the centre (postOP admission: NonTXA = 9 ± 4d, TXA = 8 ± 3, *p* = 0.039).

No complications which could be related to administration of TXA occurred. No thrombo-embolic events or seizures were observed. In the TXA group, one patient died of aspiration pneumonia. In the control group, two elderly patients experienced a continued deterioration in their general condition, which resulted in death due to multiple organ failure.

## Discussion

TXA can reduce blood loss and need for transfusion in the context of orthopaedic surgery by reversibly blocking fibrinolysis: it is therefore recommended for endoprosthetic treatments as part of the American guidelines [29]. However, by 2020 there were only 11 high-quality studies on the treatment of pertrochanteric fractures, in which TXA was used in 596 patients. These studies were summarised as part of a meta-analysis by Yu [30]. There were only 6 studies, with a total of 261 patients, in which patients were administered TXA and osteosynthesis was carried out using an intramedullary nail. Yu concluded that TXA is safe and effective, but further studies with larger sample sizes are needed. Scientific interest around the globe in reducing blood loss in cases of hip fracture by administering TXA can be seen reflected in the fact that in 2021 there were a further 38 reviews and original articles on Medline on the use of TXA in the treatment of hip fractures.

In this retrospective study, we were able to include 147 patients who received TXA preoperatively as part of treatment for a pertrochanteric femoral fracture using an intramedullary nail. Both significantly lower blood loss and a reduction in the rate of transfusions could be demonstrated. It is striking that this effect can only be identified in one of the two study sites. Whilst in Clinic 1 a significant effect of TXA on blood loss and transfusion frequency could be demonstrated, no significant differences were observed in any of the measured parameters in Clinic 2. To what extent this is due to the slightly higher ASA score and the more frequent use of aspirin cannot be determined with any certainty. Whether the use of aspirin during elective total hip or knee replacement leads to increased blood loss or greater postoperative bleeding has been investigated in several studies, and no differences have been found [31, 32]. Very disparate data can be found in the literature with respect to perioperative blood loss. For example, Tengberg et al. report blood loss at 2100.4 ml without TXA [9], whilst Chen calculate it at 616.4 ml [33]. However, blood loss where there was no administration of TXA did not differ between our two study centres and can be regarded as moderate compared to other studies (blood loss: Clinic 1 = 1.02 ± 0.46 l, Clinic 2 = 1.10 ± 0.47 l; *p* = 0.924). The patient populations of the two centres were also very similar and were distributed in a homogeneous manner, particularly within each centre. In addition, the same surgical procedure was used and the same guidelines for transfusion were followed with respect to the indication for transfusion [34]. Both clinical sites are under the same medical management and operate two-way exchanges of surgeons for the purpose of standardising surgical procedures (amongst other things), such that differences in treatment of pertrochanteric fractures between the two centres cannot be assumed to be a cause. There are only a few studies in the literature in which TXA does not lead to a reduction in perioperative blood loss. In contrast with the results from most other studies, Virani et al. were not able to identify a reduction in blood loss when applying TXA topically [35]. The cause for this remains unclear, as can also be said for our study. It is clear to see that there are factors which have not yet been identified that have an effect on the efficacy of TXA and which have not been sufficiently characterised in clinical trials.

Compared to other studies, the transfusion rate without TXA was relatively low at 31%. In this regard, Schiavone et al. 2018 investigated the treatment of pertrochanteric fractures with an intramedullary nail, and were able to reduce the transfusion rate with administration of TXA from 60.46% to 42.55% [36], i.e. even with TXA, the transfusion rate was still 11.55% higher than in our study without TXA. One of the reasons for this could be that many studies have reported a significantly lower threshold for a transfusion protocol to be triggered than is the norm in Germany. Similarly high transfusion rates were reported by Lei et all (2017), who transfused 56.09% of patients without TXA and 28.20% of patients receiving TXA [37]. With TXA, the transfusion rate in our study was a mere 20%; and in Clinic 1 it was even lower at 10%. As such, the study was able to demonstrate that even with a transfusion rate that is initially relatively low, a further reduction can be achieved through administration of TXA.

Despite both centres operating according to the cross-sectional haemotherapy guidelines from 2014 [34], there is the possibility of bias occurring when evaluating the transfusion rates due to the different assessments of the relative threshold triggers for transfusion as made by the treating physicians.

With respect to trauma patients, the CRASH II study was able to show that the shorter the time delay from trauma to administration, the greater the effectiveness of TXA [38]. For elective surgeries, TXA is given just before the start of surgery and any blood losses only occur either during the operation or after the operation. Studies with large sample sizes were able to show evidence of a benefit for patients undergoing elective procedures for endoprosthetics [18]. In the case of pertrochanteric fractures, the fracture itself leads to a significant amount of blood loss [39], which was not recorded separately in this study. The effects of tranexamic acid can only be expected to be reflected in the blood loss caused by the surgery and any postoperative blood loss. In this respect, it seems logical that improved effects would be seen in interventions involving higher blood losses, such as ORIF for pertrochanteric femoral fractures, and this could also be shown in this study.

The duration of postoperative admission following treatment of hip fractures is determined primarily on the basis of availability of geriatric rehabilitation beds and beds in short-term care facilities, with a lesser role played by actual medical grounds. In this respect, this variable should be interpreted with caution.

Determining the optimal dose of TXA is still problematic. Different dosages and timings for administration of TXA have been described in the literature. For example, a single 1 g dose has been given before surgery [35, 40, 41]; or 10 mg/kg [42] or 15 mg/kg [7, 36, 43, 44] doses have been applied according to body weight. In some cases, a second dose was administered postoperatively [9, 42, 43]. Local application of 2 g or 3 g TXA has also been trialled [35, 45]. In all of the studies cited here, blood loss associated with surgical treatment of hip fractures was reduced, and complications were not seen to increase. However, the number of cases is so small that it is not possible to make recommendations for a therapy regimen. We selected the 1 g dose primarily because it was the easiest to dose and falls within the range stated in the specialist information, which recommends 0.5 to 1 g. The dose was therefore between 10 mg/kg and 15 mg/kg for most patients.

## Conclusions

In summary, the administration of TXA prior to surgical treatment of pertrochanteric fractures with an intramedullary nail leads to a significant reduction in blood loss and the number of units of blood required for transfusion. This effect is even more pronounced in cases where higher blood losses are seen in treatment without TXA. Patient's on aspirin as a pre-existing medication may exhibit a competing effect with the TXA effect. Side effects and increased complication rates, thrombo-embolic events in particular, have not been observed. A trend towards a reduction in the frequency of postoperative renal failure can be seen, but is not significant in this study. Based on positive experiences when implemented in other surgeries with an increased risk of bleeding and based on recommendations from the current literature, routine administration of tranexamic acid as part of treatment of hip fractures should be considered, whereby ideally prospective randomised trials with significantly higher case numbers, or registry studies, would be implemented for further investigation. The effects of accidental administration of TXA on fracture-associated haemorrhage should also be investigated in further studies.

### Limitations of the study

The study included patients from two different centres. The inclusion period was relatively protracted: 4 years. However, the statistical evaluation demonstrated that the patient populations were comparable despite this. The transfusion protocol was implemented in both clinics according to the cross-sectional haemotherapy guidelines from 2014 [34], and there were no changes to the protocol over the course of the inclusion period.

This is a retrospective, case–control study (evidence level 3) with a small sample size. The patient groups from both sites differ significantly with respect to frequency of preoperative aspirin intake and ASA score, which places constraints on how we can interpret our data. Nevertheless, the study has provided an indication of the potential significance of the competing effects of ASA and the extent of comorbidities.

## Data Availability

The datasets generated during and analysed during the current study are available from the corresponding author on reasonable request.

## Abbreviations

aPPT: Activated partial thomboplastin time
BMI: Body mass index
Crea: Creatinine
CRIF: Closed reduction and internal fixation
Fig: Figure
GFR: Glomerular filtration rate
Hb: Haemoglobin
Hct: Haematocrit
i.v.: Intravenous
MANOVA: Multivariate analysis of variance
nonTXA: Group patient group got no tranexamic acid
ORIF: Open reduction and internal fixation
postop: Postoperative
preOP: Preoperative
RCC: Red cell concentrate
SOP: Standard operating procedure
TXA group: Patient group got tranexamic acid
TXA: Tranexamic acid
